# Hydrostatic Pressure and Temperature Effects on the Membranes of a Seasonally Migrating Marine Copepod

**DOI:** 10.1371/journal.pone.0111043

**Published:** 2014-10-22

**Authors:** David W. Pond, Geraint A. Tarling, Daniel J. Mayor

**Affiliations:** 1 Scottish Association for Marine Science, Oban, Argyll, United Kingdom; 2 British Antarctic Survey, Natural Environment Research Council, Cambridge, United Kingdom; 3 Institute of Biological and Environmental Sciences, Oceanlab, University of Aberdeen, Newburgh, Aberdeenshire, United Kingdom; Stazione Zoologica Anton Dohrn, Naples, Italy

## Abstract

Marine planktonic copepods of the order Calanoida are central to the ecology and productivity of high latitude ecosystems, representing the interface between primary producers and fish. These animals typically undertake a seasonal vertical migration into the deep sea, where they remain dormant for periods of between three and nine months. Descending copepods are subject to low temperatures and increased hydrostatic pressures. Nothing is known about how these organisms adapt their membranes to these environmental stressors. We collected copepods (*Calanoides acutus*) from the Southern Ocean at depth horizons ranging from surface waters down to 1000 m. Temperature and/or pressure both had significant, additive effects on the overall composition of the membrane phospholipid fatty acids (PLFAs) in *C. acutus*. The most prominent constituent of the PLFAs, the polyunsaturated fatty acid docosahexanoic acid [DHA – 22:6(n-3)], was affected by a significant interaction between temperature and pressure. This moiety increased with pressure, with the rate of increase being greater at colder temperatures. We suggest that DHA is key to the physiological adaptations of vertically migrating zooplankton, most likely because the biophysical properties of this compound are suited to maintaining membrane order in the cold, high pressure conditions that persist in the deep sea. As copepods cannot synthesise DHA and do not feed during dormancy, sufficient DHA must be accumulated through ingestion before migration is initiated. Climate-driven changes in the timing and abundance of the flagellated microplankton that supply DHA to copepods have major implications for the capacity of these animals to undertake their seasonal life cycle successfully.

## Introduction

Aquatic environments are characterised by gradients of temperature and pressure [depth], both of which affect the functioning of biological membranes. Phospholipids are major constituents of cellular membranes and their fatty acid composition is regulated to maintain membrane order, otherwise termed, membrane ‘fluidity’ [1]. The maintenance of membrane order and a liquid-crystalline state is of critical importance for the function and integrity of cell membranes, since it controls bilayer permeability and also the mobility and function of embedded enzymes [2]. Typical responses to both increasing pressure and decreasing temperature are to increase the proportions of unsaturated fatty acids in the membranes, a process termed homeoviscous adaptation (HVA; [1], [3]–[4]).

Most work on adaptive changes in the lipid bilayers of poikilotherms has been conducted on strains of deep sea bacteria which are amenable to experimental manipulation. These organisms increase the proportions of mono- and polyunsaturated fatty acids in their membrane phospholipids in response to increased pressure and/or reduced temperature [5]–[7]. A comparison of mitochondrial membranes isolated from shallow and deep sea fish species has similarly identified changes in unsaturation that are consistent with pressure dependent HVA [8]–[9]. Pressure induced membrane adaptations have largely been considered independently to temperature effects [10]. However, vertical gradients in the oceans frequently encompass changes in both temperature and pressure. Calanoid copepods, which dominate marine zooplankton biomass at latitudes greater than 30° north and south [11], undergo a migration to depth during winter [12]–[13] in what is arguably the largest seasonal migration on Earth. These animals may encounter a 10°C and ∼350 bar change in temperature and pressure respectively over the course of only a few days or weeks, providing a natural system for studying the functional responses of lipid bilayers to these environmental stressors.

**Figure 1:**
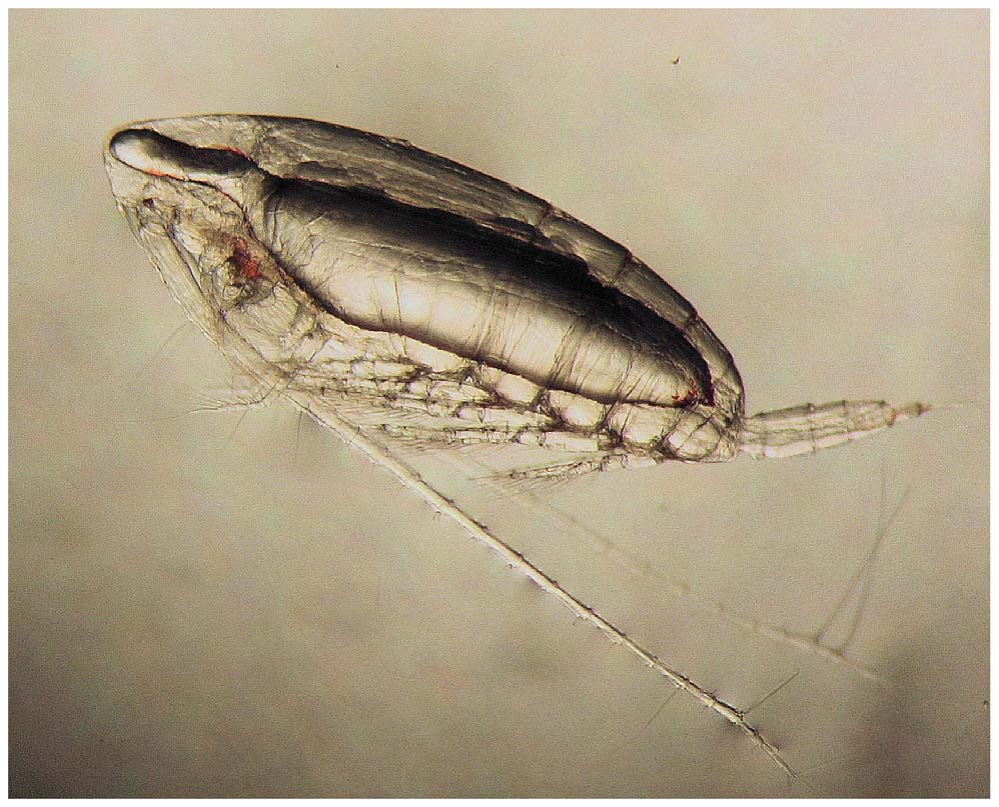
*Calanoides acutus*, a marine copepod that descends into the deep sea to overwinter in a state of dormancy. The large oil sac occupying the abdomen is thought to provide energy storage and aid buoyancy control. Body length 3 mm.

This study examined the overarching hypothesis that temperature and pressure affect the composition of fatty acids in the membrane phospholipids of the dominant large calanoid copepod in the Southern Ocean, *Calanoides acutus* (Fig. 1). These animals descend to 500–3500 m each austral summer to overwinter winter in a state of dormancy [14]. During late winter, the copepods ascend to the upper ocean to mature into adults and commence feeding and reproductive activity.

## Methods

### Specific permissions are not required for sampling of marine invertebrates

#### Sample collection

**Figure 2:**
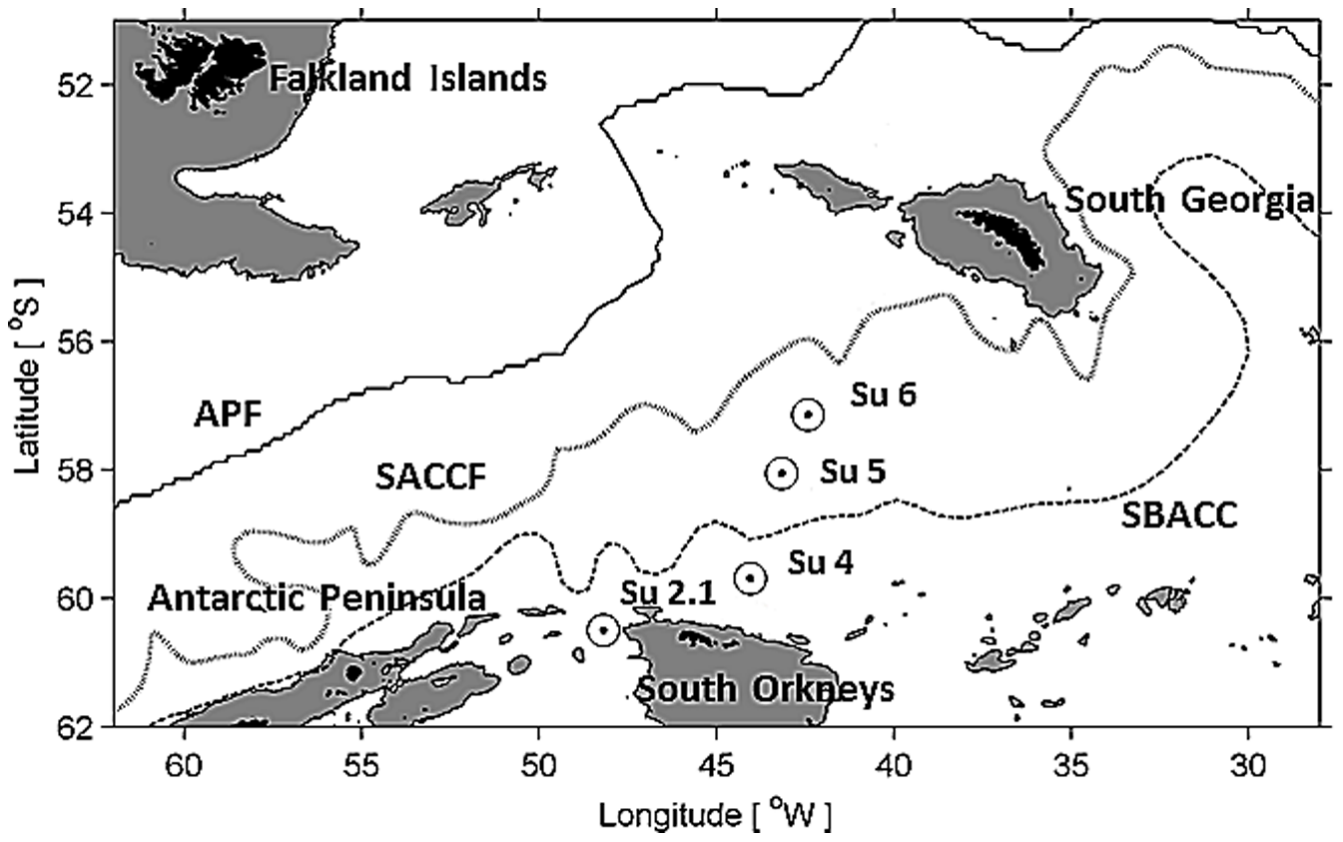
Map indicating locations of the four study sites in the Southern Ocean. (APF = Antarctic Polar Front, SACCF = Southern Antarctic Circumpolar Current Front, SBACC = Southern Boundary of the Antarctic Circumpolar Current).

**Figure 3:**
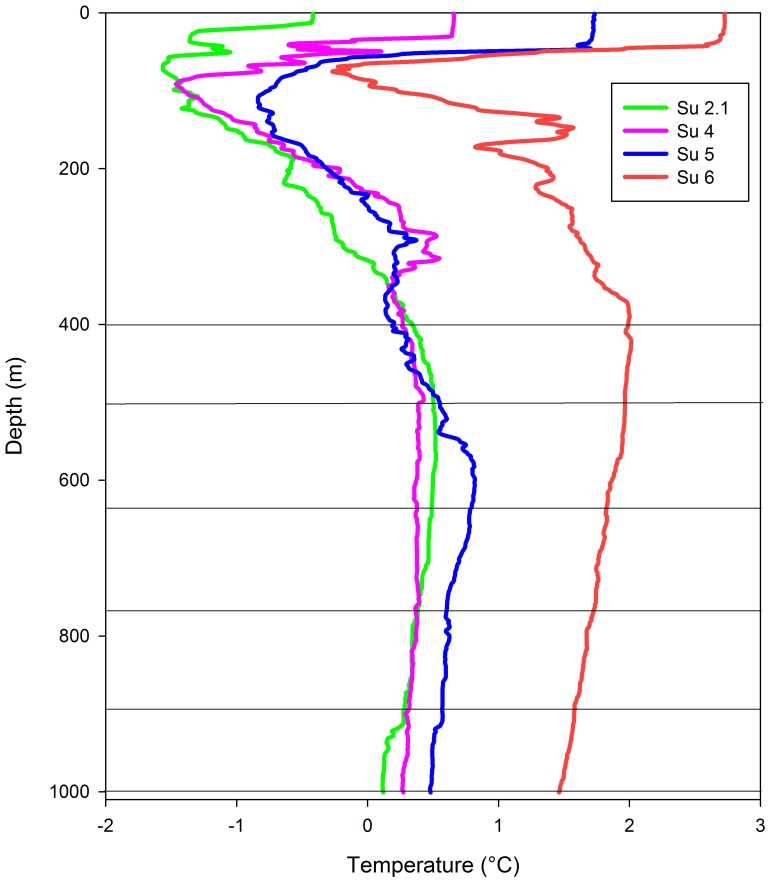
Profiles of temperature at the four biological sampling stations in the Scotia Sea (Jan–Feb 2008). Horizontal lines depict the sampling intervals of the Bongo net (0–400 m) and the MOCNESS multinet (375–1000 m).

Copepods were collected from four stations during a cruise of the RRS James Clark Ross to the Scotia Sea in January and February 2008 (Fig. 2, Table S1, [15]–[16]). Vertical profiles of pressure are linear and effectively constant between stations whereas temperature profiles, determined at dawn using a SeaBird 911+CTD, differed between stations (Fig. 3). This sampling design enabled us to reliably distinguish between the effects of pressure and temperature; for any given pressure we had observations across a range of temperatures. Stratified samples were collected during the night from 375 m to 1000 m in 125 m depth horizons using a MOCNESS multi-net. Copepods from the photic zone water column were collected using Bongo nets deployed immediately after the CTD deployments and were hauled vertically at ∼0.22 ms^−1^ from 400 m to the surface [16]. A total of 85 individual *C. acutus* (pre-adult 5^th^ copepodite stage) were collected over four stations and six depth horizons. Copepods were individually sorted onboard and stored in 500 µl of chloroform∶methanol (2∶1 v/v) in 1.1 ml tapered vials (Chromacol) at −80°C until analysis. All samples were analysed in laboratories in Cambridge <1 year after sample collection.

#### Lipid analysis

Total lipid was extracted following Folch et al. [17]. Given the comparatively small amounts of lipid contained within an individual copepod, extraction procedures were simplified to reduce losses and minimise the introduction of contaminants into the samples [15]. After the addition of 125 mL of KCl (0.88% w/v), samples were initially whirlimixed before being centrifuged for 2 min at 1500 rpm to promote phase separation. The lower chloroform phase, containing the total lipid extract was removed using a 500 mL Hamilton glass syringe equipped with a teflon tipped plunger. The needle of the syringe was pushed through the upper methanol–water layer and the chloroform phase taken up into the syringe, leaving the aqueous phase as waste in the original vial. Tapered vials greatly facilitate this procedure. Phospholipids, which are major constituents of membrane lipids were purified from total lipid using high performance thin layer chromatography (HPTLC) in conjunction with a 18∶2∶0.2 (hexane/diethyl ether/acetic acid, v/v/v) solvent system. Phospholipids were then transesterified to generate fatty acid methyl esters and analysed on a TRACE 2000, Thermo Electron gas chromatograph (GC). The GC was equipped with oncolumn injection, a Stabilwax column (Restek 30 m×0.32 mm internal diameter) and hydrogen was used as the carrier gas. Data are expressed as percent composition of total fatty acids. See Purać et al. [18] for full details of the methods used.

#### Data analysis

Preliminary data exploration was undertaken to identify outliers and instances where explanatory variables were highly correlated [19], [20]. The effects of depth (mid-sampling depth), temperature and station identity on the percentage composition of phospholipid fatty acids (PLFAs) in *C. acutus* were investigated using redundancy analysis (RDA).

The significance of each model term was assessed using a permuted (n = 9999) forwards selection procedure [19]. Linear regression techniques were used to examine how depth, temperature and a depth×temperature interaction affected the percentage abundance of the major (mean>5%) PLFAs in *C. acutus*. Where necessary, station identity was included as a random effect, thereby accounting for correlations between observations made within each sampling station. Variance covariates were also included in the random structure of the regression models where problems with unequal variance were identified. The procedures for model selection using generalised least squares- (GLS) and linear mixed-effects (LME) regression models are described elsewhere [20]–[22]. In brief, the random structures of the statistical models were established using a likelihood ratio (L. ratio) test using restricted maximum likelihood (REML) estimation. The fixed structure of each model was subsequently determined using a hierarchical backwards selection procedure based on the L. ratio test using maximum likelihood estimation. Model parameters were generated using REML estimation. The underlying statistical assumptions were verified as follows: theoretical quantiles were plotted against standardized residuals (Q-Q plot) to assess normality of residuals; residual values were plotted against fitted values and each covariate to verify homogeneity of variance and independence of observations respectively. Statistical modelling was conducted in the ‘R v2.11.1’ programming environment [23] using the ‘vegan’ [24] and ‘nlme’ [25] packages.

## Results

### Oceanography

Sampling stations covered a latitudinal range of around 5° and water depths of between 1400 m and 2700 m. Two of the stations (Su 2.1 and Su 4.0) lay south of Southern Boundary in the coldest Southern Ocean water mass. Station Su 5.0 lay north of this front but south of the Southern Antarctic Circumpolar Current Front (SACCF) while Su 6.0 was located just north SACCF. Near-surface temperatures were at least a whole degree colder in the two southerly stations compared to the two northerly stations (−0.5°C to −0.6°C compared to 0.5°C to 1.4°C, Fig. 3). Nevertheless, the vertical water mass structure below the surface was relatively similar across all sampling stations. Temperatures initially declined steeply until reaching a temperature minimum at around 100 m depth (−1.4°C in the south, 0°C to the north). This decline is the product of old winter water that lies beneath Antarctic surface waters. With greater depth, temperature increased through the mixing of winter water with warmer upper circumpolar deep water. No further temperature increase occurred below 500 m after which there was only a very gradual decline in temperature until the maximum sampling depth. Overall, after experiencing some variation in temperature while descending through the upper 400 m of the water column, a change of less than 0.4°C is experienced through the remainder of the water column in any one location. Between locations, there was a variation of around 1.5°C at comparative depths between the most southerly and northerly sampling stations (Fig. 3).

### Membrane fatty acids

The PLFAs of 85 individual copepods were analysed in this study. These animals were sampled from 4 stations across temperature and depth gradients of 2.5°C and 1000 m respectively. A summary of the PLFA data is presented in Table 1. The polyunsaturated (PUFA) fatty acid docosahexaenoic [DHA; 22:6(n-3)] dominated the PLFAs of *C. acutus*, constituting up to 46.1% (28.7±0.8%; mean ± SE). Other fatty acids that contributed significantly (>5% of mean values) to the phospholipids were the PUFA eicosapentaenoic acid [EPA; 20:5(n-3)] (18.9±0.6%) and the saturated fatty acids (SFAs) palmitic acid [16:0] (18.6±0.3%) and stearic acid [18:0] (6.6±0.6%).

**Table 1:** Average fatty acid composition of phospholipids in *C. acutus* from 4 stations (n = number of individual copepods sampled from each station, * position of double bonds uncertain).

Fatty acid	Station									
	2.1 (n = 24)	(SE)	4 (n = 30)	(SE)	5 (n = 13)	(SE).	6 (n = 18)	(SE).	Overall mean	(SE)
14:0	3.8	(0.3)	3.4	(0.3)	2.0	(0.2)	2.9	(0.2)	3.2	0.2
15:0	0.4	(0.1)	0.4	(0.1)	0.3	(0.1)	0. 7	(0.1)	0.4	0.1
16:0	17.2	(0.6)	17.7	(0.5)	20.8	(0.8)	20.5	(0.4)	18.6	0.3
16:1(n-7)	1.8	(0.2)	1.8	(0.1)	1.6	(0.1)	1.8	(0.1)	1.8	0.1
16:2(n-4)	5.9	(1.0)	3.8	(0.7)	2.0	(0.3)	8.0	(0.7)	5.0	0.4
18:0	5.2	(0.6)	3.8	(0.3)	4.3	(0.5)	15.0	(1.3)	6.6	0.6
18:1(n-9)	4.2	(0.2)	3.6	(0.4)	3.8	(0.3)	3.2	(0.2)	3.7	0.1
18:1(n-7)	2.8	(0.1)	2.7	(0.1)	2.4	(0.2)	2.2	(0.35)	2. 6	0.1
18:1(n-5)	5.1	(0.3)	4.2	(0.2)	4.2	(0.3)	5.6	(0.4)	4.7	0.3
18:2(n-6)	0.9	(0.1)	1.0	(0.1)	0.7	(0.04)	1.4	(0.4)	1.0	0.1
18:4(n-3)	0.3	(0.1)	0.2	(0.1)	0.6	(0.1)	0.3	(0.03)	0.3	0.03
20:1(n-9)	1.4	(0.1)	1.3	(0.1)	1.2	(0.1)	0.4	(0.1)	1.1	0.1
20:4(n-6)	0.1	(0.02)	0.4	(0.1)	0.3	(0.1)	0.1	(0.04)	0.2	0.03
20:4(n-3)	0.8	(0.1)	0.6	(0.1)	0.5	(0.1)	0.4	(0.1)	0.6	0.1
20:4*	1.3	(0.5)	1.8	(0.6)	1.2	(0.1)	0. 5	(0.1)	1.3	0.3
20:5(n-3)	17.3	(1.1)	20.8	(0.8)	24.8	(0.7)	13.8	(0.9)	18.9	0.6
22:1(n-11)	0.2	(0.03)	0.2	(0.03)	0.3	(0.1)	0.4	(0.1)	0.2	0.04
22:1(n-9)	1.0	(0.2)	0.6	(0.1)	0.6	(0.1)	0.2	(0.1)	0.6	0.1
22:5(n-3)	0.3	(0.1)	0.7	(0.1)	1.0	(0.1)	0.2	(0.03)	0.5	0.1
22:6(n-3)	30.2	(1.8)	31.4	(1.2)	27.7	(1.3)	23.1	(1.0)	28.7	0.7

**Figure 4:**
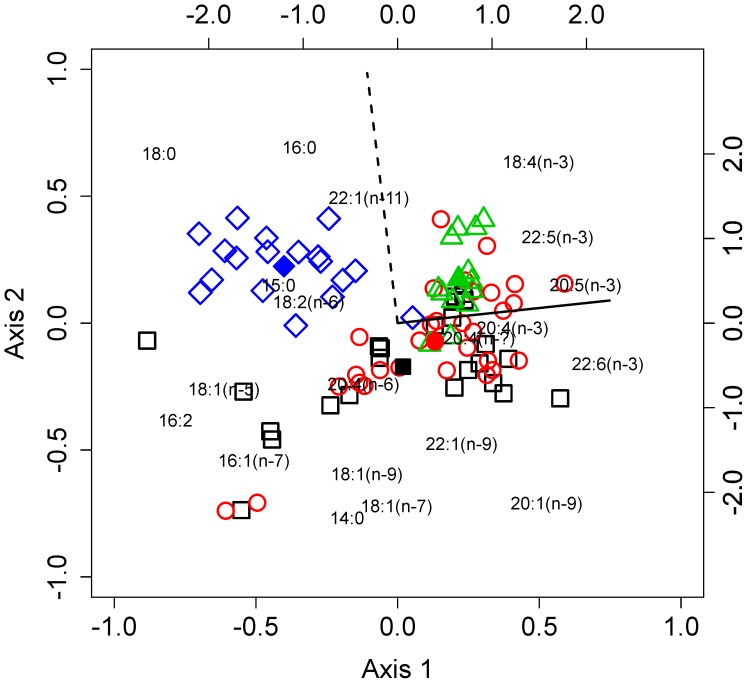
Redundancy analysis distance triplot of the phospholipid fatty acid data from 85 individual *C. acutus* (‘sites’) using 20 fatty acids (‘species’). The effects of depth and temperature are plotted as vectors (solid- and dashed lines respectively). Symbols and colours denote Station identity, with the overall effect of each Station being represented by filled symbols. The primary and secondary sets of axes relate to the sites and species loadings respectively.

In order of importance, depth (F = 11.34, df = 1, p<0.001), temperature (F = 7.29, df = 1, p<0.001) and station identity (F = 5.18, df = 3, p<0.001) all increased the amount of variance explained in the PLFA compositional data. These explanatory variables accounted for 32% of the variation in the data and 26% was explained by the first two axes of the redundancy analysis. The RDA triplot visualises how the explanatory variables influence the composition of PLFAs (Fig. 4). The first axis, which was positively correlated with depth, had strong positive loadings of the PUFAs EPA and DHA and negative loadings of the SFAs 18:0 and 16:0. The second axis had strong positive loadings of the SFAs 16:0 and 18:0 and was positively correlated with temperature.

**Figure 5:**
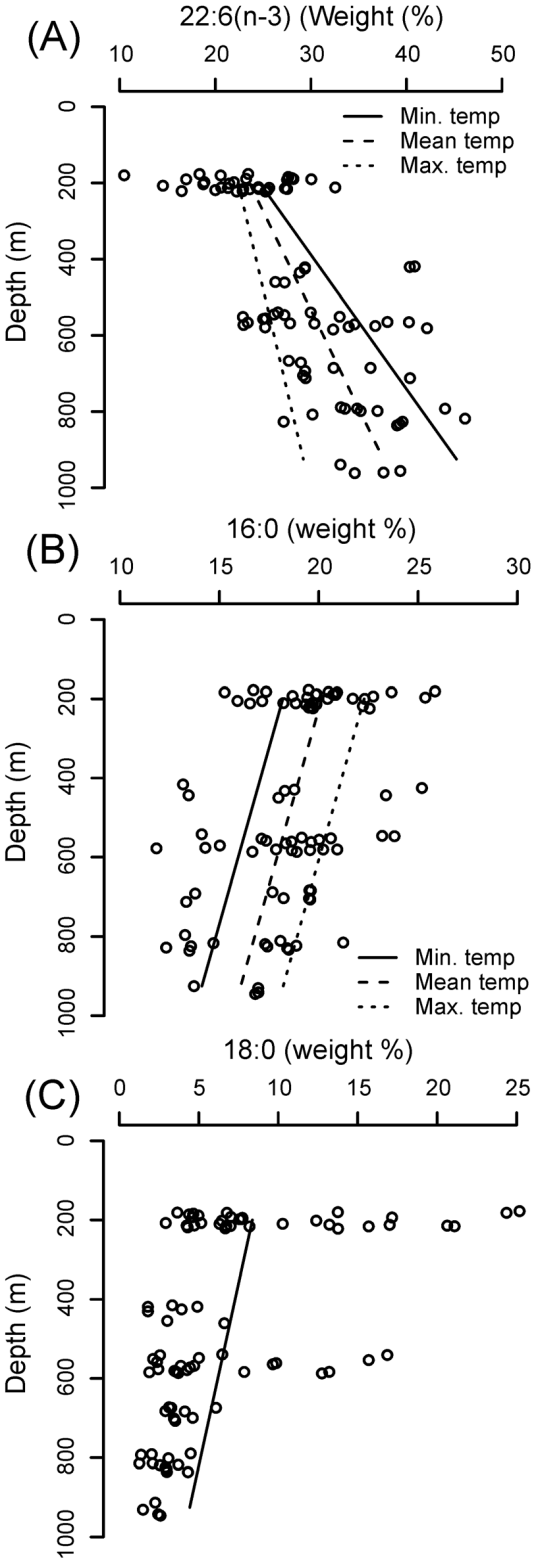
Model-predicted effects of depth and temperature on the percentage composition of (a) 22:6(n-3), (b) 16:0 and (c) 18:0 in the phospholipid fatty acids of *C. acutus*. Data points are presented for guidance only.

**Figure 6:**
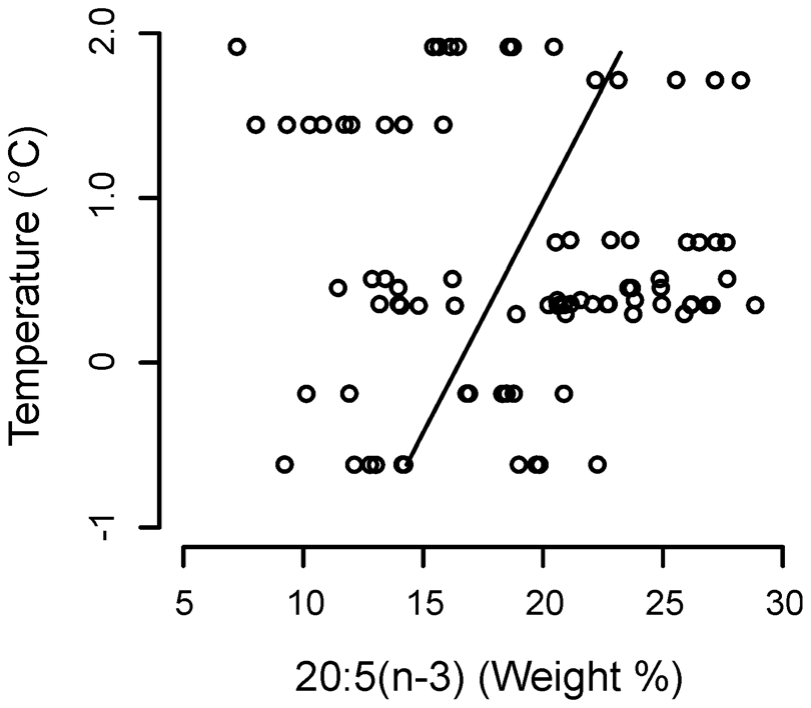
Model-predicted effects of temperature on the percentage composition of 20:5(n-3) in the phospholipid fatty acids of *C. acutus*. Data points are presented for guidance only.

The PLFAs DHA, 16:0 and 18:0 were all significantly affected by depth (Tables 2–5). The PUFA DHA increased with depth, whereas the SFAs 16:0 and 18:0 both decreased (Figure 5 A–C; Tables 2–5). In the case of DHA, the effects of depth were dependent upon the effect of temperature; the effect of depth on this fatty acid increased as temperatures decreased (Fig. 5A; Tables 2 and 3). Temperature had a significant, additive effect on the SFA 16:0 (Fig. 5B; Tables 2 and 4); at any given depth, the percentage abundance of this compound increased as temperature increased (Fig. 5C). The PUFA EPA was not affected by depth (L. Ratio = 1.853, df = 1, p = 0.174) but increased significantly with temperature (Fig. 6; Tables 2 and 6).

**Table 2:** Summary of fixed-, random^(†)^ and variance covariate^(‡)^ terms in the optimal statistical models.

Response	Model term	df	L. ratio	p
22:6(n-3)	Depth×Temperature	1	5.590	0.018
	Temperature^‡^	1	5.561	0.018
16:0	Depth	1	24.145	<0.001
	Temperature	1	19.167	<0.001
18:0	Depth	1	39.619	<0.001
	Station^†^	1	56.278	<0.001
	Station^‡^	3	35.387	<0.001
20:5(n-3)	Temperature	1	8.479	0.004
	Station^†^	1	33.612	<0.001

**Table 3:** The effects of depth and temperature on the percentage composition of 22:6(n-3) in the phospholipids of *C. acutus*.

Model parameter	Coefficient (± SE)	t	p
Intercept	19.341±1.607	12.038	<0.001
Depth	0.024±0.003	7.821	<0.001
Temperature	0.608±1.388	0.438	0.663
Depth×Temperature	−0.008±0.003	−2.343	0.022

**Table 4:** The effects of depth and temperature on the percentage composition of 16:0 in the phospholipids of *C. acutus*.

Model parameter	Coefficient (± SE)	t	p
Intercept	20.313±0.612	33.186	<0.001
Depth	−0.006±0.001	−5.190	<0.001
Temperature	1.613±0.354	4.552	<0.001

**Table 5:** The effect of depth on the percentage composition of 18:0 in the phospholipids of *C. acutus*.

Model parameter	Coefficient (± SE)	t	p
Intercept	9.463±2.437	3.883	<0.001
Depth	−0.005±0.001	−7.215	<0.001

**Table 6:** The effect of depth on the percentage composition of EPA (20:5(n-3)) in the phospholipids of *C. acutus*.

Model parameter	Coefficient (± SE)	t	p
Intercept	16.519±3.116	5.3018	<0.001
Temperature	3.567±1.114	3.138	0.0024

## Discussion

Our data demonstrate that the composition of fatty acids in the membranes of a seasonally migrating copepod, *C. acutus*, is influenced by a range of factors, including depth, temperature and geographic location. We found that the membrane phospholipids of these animals are dominated by DHA, with the proportions of DHA increasing with depth. This effect is exacerbated at colder temperatures. Such an interaction between temperature and pressure is entirely consistent with homeoviscous adaptation theory (HVA) theory, and is the first time that such a relationship has been demonstrated in a metazoan organism.

Knowledge of pressure and temperature adaptations in biological membranes has largely been derived from deep-sea bacteria [5]–[6], [26]–[27]. Monounsaturated fatty acids (MFAs) are most commonly reported as the agents involved in maintaining membrane order, with the addition of further double bonds having little effect on the phase transition properties and hence fluidity of the lipids [28]. The predominance of DHA in the membranes of *C. acutus* indicates that this PUFA is a central part of their adaptive response to the effects of both temperature and pressure on membrane functionality. DHA is amongst the most unsaturated fatty acids found in nature. It contributes to the hyperfluidization of membranes and mediates processes catalysed by membrane proteins [7], [29]. We therefore hypothesize that, in addition to maintaining membrane fluidity, DHA confers temperature and pressure tolerance to other essential properties of membrane function that are linked to the activity of membrane proteins and metabolism [29].

Calanoid copepods cannot synthesize DHA de novo [30] and this fatty acid is therefore considered to be essential, i.e. it must be derived from their diet. Flagellated microplankton are the predominant producers of DHA in the marine environment [31] and are likely to be the ultimate source of this compound in *C. acutus*. However, seasonally migrating copepods do not feed, but they sequester large lipid reserves within an internal sac during the spring and summer plankton blooms and later utilise this as an energy reserve whilst undertaking their seasonal migration (Fig. 1). The lipid reserve of *C. acutus* contains appreciable quantities of DHA [16], and is most likely the source of fatty acids used for maintaining membrane order during migration. Despite the fatty acid composition of biological membranes being tightly regulated, we found geographical differences in the phospholipid fatty acid signatures of *C. acutus* (Fig. 4). This probably reflects differences in microplankton communities at the different locations [16].

The composition of lipids in the large oil sac was previously thought to reflect the accumulation of dietary lipids with little control on its composition by the copepod. More recent evidence suggests that the composition of the lipid sac is regulated since this determines its solid-liquid phase transition and enables the copepod to achieve neutral buoyancy in the deep sea [15], [32]. Neutral buoyancy is essential if the copepods are to minimise swimming activity, which would attract predators and consume valuable energy reserves during the period of non-feeding. EPA is thought to play a key role in modulating the response of the oil sac to pressure and hence the extent to which this sac provides hydrostatic lift [15], [32]). EPA is an essential dietary component [30] and ultimately derived from diatoms [15]. Collectively, these findings indicate two major functional roles for fatty acids derived from two distinct algal groups. EPA from diatoms is used to regulate buoyancy and the depth at which neutral buoyancy is achieved whilst DHA from flagellated microplankon is used to maintain biological membrane function. Climate-driven phenological change in the plankton, which is causing a temporal mismatch between zooplankton and the availability of their phytoplankton prey [33] therefore not only has implications for trophic transfer, but also for the successful overwintering of seasonally migrating copepods.

The additive effects of temperature and/or depth on the fatty acids 16:0 and 18:0 reported herein are consistent with the understanding that organisms manipulate the level of unsaturation of their membranes in order to maintain membrane functionality. Indeed, the inverse relationship between the PUFA DHA and SFAs (16:0 and 18:0) suggests that *C. acutus* respond to changes in their physical environment largely by changing the relative proportions of these compounds. It is possible that 16:0 and 18:0 are used interchangeably, as these compounds were similarly affected by pressure in the current study. However, the additional, additive effect of temperature on 16:0 suggests that the biophysical properties of this compound may differ to those of 18:0. The insignificant effect of depth on EPA is not entirely surprising given recent observations which suggest that this PUFA is not necessarily required for maintaining membrane fluidity, but rather plays a beneficial role in cell division and membrane organisation [34]. Indeed, the central role of EPA in buoyancy regulation may necessitate that this compound is only used for membrane maintenance when sufficient DHA is not immediately available. This interpretation is also consistent with the conformational properties and functions of EPA and DHA [35] and may explain why we did not find interactive effects between temperature and depth for EPA. Further investigation is required to address this more fully.

Membranes of mammals and birds also contain high proportions of DHA which co-vary with the MFA 18:1(n-9) [29]. In the copepods 18:1(n-9) was a minor component of PLFAs, and was substituted with the SFAs 16:0 and 18:0. These differences in the unsaturation of membrane bilayer composition between birds, mammals and invertebrates could plausibly be linked to metabolic activity [2]. Extrapolation of data in Fig. 5A suggests that *C. acutus* could potentially reach 3,000 m before its phospholipids would solely constitute DHA. This seems unlikely given that such high levels of DHA in biological membranes have not previously been found in nature. Teleosts have been reported at 8,000 m, and crustaceans are well known to thrive at full ocean depth, of around 11,000 m [36]. These observations apparently confirm that additional and/or alternative strategies for the maintenance of membrane functionality must be in existence and require further investigation. A study of cold tolerance and phospholipid saturation in the nematode, *Caenorhabditis ele*gans, concluded that lipid saturation accounted from only 16% of observed differences in a variety of parameters for animals held at 10°C and 25°C. [37]. At the level of the whole organism, adaptation to pressure and thermal stressors is likely to be complex, with the unsaturation of membrane phospholipids being just one aspect, although it is clear that DHA plays an important role in the adaptation to pressure of *C. acutus* during its extensive vertical migrations through the ocean.

The ability to adapt to pressure suggests the existence of a mechanism by which copepods actually sense pressure. Some calcified crustaceans utilise statocysts to detect hydrostatic pressure [38]. Rudimentary statocysts are also present in calanoid copepods [39], although their potential role in the sensing of pressure is not known. Several pressure-regulated operons have been reported in deep-sea bacteria that regulate the synthesis of proteins that confer barotolerance, yet our understanding of the controlling mechanisms remains limited [40]. Clearly there is much to be learnt about the mechanism(s) through which aquatic organisms sense and respond to pressure.

We have demonstrated that DHA plays an important adaptive role in the life cycle of a species of marine copepod that undertakes extensive vertical migrations, and hence experiences changes in environmental temperature and pressure. The majority of DHA in the marine environment originates in flagellated microplankton, which must be accumulated in sufficient quantities via the diet before migration is initiated. Future, climate-driven changes in oceanic productivity and microplankton species composition could have major implications for the capacity of these animals to complete their seasonal life cycle.

## Supporting Information

Table S1 (XLSX)
